# RepB C-terminus mutation of a pRi-*repABC* binary vector affects plasmid copy number in *Agrobacterium* and transgene copy number in plants

**DOI:** 10.1371/journal.pone.0200972

**Published:** 2018-11-09

**Authors:** Zarir Vaghchhipawala, Sharon Radke, Ervin Nagy, Mary L. Russell, Susan Johnson, Stanton B. Gelvin, Larry A. Gilbertson, Xudong Ye

**Affiliations:** 1 Monsanto Company, St. Louis, MO, United States of America; 2 Woodland Campus, Monsanto Company, Woodland, CA, United States of America; 3 Department of Biological Sciences, Purdue University, West Lafayette, IN, United States of America; Academia Sinica, TAIWAN

## Abstract

A native *repABC* replication origin from pRiA4b was previously reported as a single copy plasmid in *Agrobacterium tumefaciens* and can improve the production of transgenic plants with a single copy insertion of transgenes when it is used in binary vectors for *Agrobacterium*-mediated transformation. A high copy pRi-*repABC* variant plasmid, pTF::Ri, which does not improve the frequency of single copy transgenic plants, has been reported in the literature. Sequencing the high copy pTF::Ri *repABC* operon revealed the presence of two mutations: one silent mutation and one missense mutation that changes a tyrosine to a histidine (Y299H) in a highly conserved area of the C-terminus of the RepB protein (RepB^Y299H^). Reproducing these mutations in the wild-type pRi-*repABC* binary vector showed that *Agrobacterium* cells with the RepB^Y299H^ mutation grow faster on both solidified and in liquid medium, and have higher plasmid copy number as determined by ddPCR. In order to investigate the impact of the RepB^Y299H^ mutation on transformation and quality plant production, the RepB^Y299H^ mutated pRi-*repABC* binary vector was compared with the original wild-type pRi-*repABC* binary vector and a multi-copy oriV binary vector in canola transformation. Molecular analyses of the canola transgenic plants demonstrated that the multi-copy pRi-*repABC* with the RepB^Y299H^ mutation provides no advantage in generating high frequency single copy, backbone-free transgenic plants in comparison with the single copy wild-type pRi-*repABC* binary vector.

## Introduction

Single copy, vector backbone-free transgene events are required by government regulatory agencies for commercial transgenic product deployment, and are preferred for many other biotechnology applications. This is because most vector backbones contain antibiotic genes and other not fully characterized sequences, and that multi-copy transgenes may have reduced or silenced gene expression [1–3]. Efforts have been made to increase single copy transgene frequency, and to eliminate the presence of vector backbone sequences in transgenic plants using particle bombardment or *Agrobacterium*-mediated transformation [4–9]. Lowering DNA loading in particle bombardment [4–8], or using a single copy binary vector rather than multi-copy vectors in *Agrobacterium* transformation [9] can significantly increase single copy transgenic plant production.

*Agrobacterium*-mediated plant transformation often uses a binary vector to maintain the plasmid in *Escherichia coli* for gene cloning manipulation and in *Agrobacterium* cells to deliver the T-strand into plant cells. Different origins of replication control the binary vector copy number in *Agrobacterium* which can further affect T-strand processing and integration into the plant genome. The broad host range plasmid RK2-derived origin of replication (RK2 oriV, *incPα* group, GenBank accession #J01780) replicates to approximately 10 copies/Agrobacterium chromosome [10], whereas the pRi-*repABC*, a plasmid with the origin of replication from the *Agrobacterium rhizogenes* plasmid pRiA4b, (hereafter denoted oriRi, GenBank accession # X04833), replicates the plasmid as 1 copy/Agrobacterium chromosome [11–13]. The oriRi plasmid contains the *repABC* operon encoding the RepA and RepB proteins for plasmid partitioning, and the replication initiator protein RepC for DNA synthesis [14,15]. A small RNA, which binds to the region of *repABC* mRNA between *repB* and *repC*, tightly regulates *repC* expression to control plasmid copy number and determines plasmid incompatibility [16,17]. RepB proteins bind specifically to *parS*-like sequences, and are required for proper segregation of replicated plasmids to daughter cells. RepB can form dimers and oligomers in solution. The central part of RepB is responsible for *parS* binding and the C-terminus determines RepB dimerization/oligomerization [18]. The putative *parS* binding site identified in the plasmid pRiA4b is 28 bp downstream from the end of the *repC* ORF [19, 20].

Native oriRi-based binary vectors produce a higher percentage of single copy, backbone free transgenic plants across multiple crops than do multi-copy binary vectors [9]. A new variant of the pRi-*repABC* replicon based vector, named pTF::Ri, has been reported with an estimated copy number 15–20 per *Agrobacterium* cell. This binary plasmid provided no advantage in single copy transgenic plant production [21].

To address the discrepancy between the results obtained with the native oriRi base vector [9] and the pTF::Ri [21] multi-copy vector, we sequenced the high copy plasmid pTF::Ri. Sequence analysis of the entire plasmid resulted in the identification of two point mutations in the *repB* gene of the pTF::Ri plasmid. In this manuscript, we characterize these two mutations in the *repB* gene of the pTF::Ri plasmid for their effect on plasmid copy number in *Agrobacterium*, and their impact on the frequency of single copy, backbone-free transgenic canola plant production.

## Materials and methods

### Plasmid sequence and construction

The high copy oriRi plasmid pTF::Ri [21] was sequenced and assembled by the Monsanto sequence center. This new sequence was aligned with the published pRiA4b sequence (GenBank accession # X04833) using with Needleman-Wunsch algorithm [22].

The oriRi plasmid pMON83937 [9], which contains 4114 bp of the *repABC* sequence from pRiA4b (GenBank X04833, between 7–4120 bp), was used for all site-directed mutagenesis experiments using the QuickChange II protocol (Agilent Cat # 200521). The primers 5’ GATCATGTGCCAGCGCTG**cAT**CAAGCGTACCACGCTG 3’ (forward) and 5’ CAGCGTGGTACGCTTG**ATg**CAGCGCTGGCACATGATC 3’ (reverse) were used to generate the RepB^Y299H^ mutation, whereas the primers 5’ GCAGTTTTCTCGAGAG**ATc**GTCATCGCCGCGATGTCG 3’ (forward) and 5’ CGACATCGCGGCGATGAC**gAT**CTCTCGAGAAAACTGC 3’ (reverse) were used to produce the *repB* T486C silent mutation. Ten ng of pMON83937 was used in a 50 μl reaction volume and amplified for 25 cycles with 5 min elongation time. The reaction mixture was incubated with *Dpn*I to remove the template and transfected into *E*. *coli* DH10B competent cells to recover the plasmids. The resultant plasmids were extracted using a Qiagen maxiprep kit and verified by full plasmid sequencing. The plasmids used for this study are summarized in Table 1.

**Table 1:** Plasmids used for this study.

Plasmid	Description of *repABC*	Estimated copy in Agrobacterium*	Reference
**pTF:Ri**	PCR amplified from pRiA4b, RepB^Y299H^ + *repB*^T486C^	15–20	Oltmanns et al. (2010)
**pMON83937**	4114 bp native *repABC*, same as GenBank X04833 between 7–4120 bp	1	Ye et al. (2011)
**pMON83937-RepB**^**Y299H**^	Site-directed RepB^Y299H^ mutation from pMON83937	9~10	This study
**pMON83937-*repB***^**T486C**^	Site-directed *RepB*^T486C^ mutation from pMON83937	1	This study
**pMON83937 RepB**^**Y299H**^ **+ *repB***^**T486C**^	Site-directed double mutations from pMON83937	9~10	This study
**pMON67438**	Non-*repABC* control, *incPα* group, RK2 oriV plasmid origin of replication	12	Ye et al. (2011)

* The plasmid copy number is estimated by ddPCR in this study except for the pTF::Ri from the original DNA blot

### *Agrobacterium* DNA extraction and plasmid copy number determination

*Agrobacterium* total DNA was prepared according to Estrella et al. [23] with modifications. Briefly, *Agrobacterium* containing the corresponding plasmids was grown in LB medium at 30°C with 50 mg/L spectinomycin selection. When the A_600_ reached approximately 1.0, 1 ml of *Agrobacterium* cells was harvested into an Eppendorf tube and centrifuged at 15000 rpm for 5 min. The pellet was suspended in 300 μl P1 buffer (from Qiagen Maxiprep kit with RNaseA), 100 μl N-lauroylsarcosine (5% solution in TE) and mixed well, followed by addition of 100 μl Pronase (Calbiochem, 2.5 mg/ml solution in TE buffer). The solution was incubated at 37°C for ~2 hours. The lysate was sheared by passing through a 1 ml pipet tip ~10 times, extracted twice with an equal volume of phenol/chloroform (pH 8), and then twice with an equal volume of chloroform. Total DNA from the aqueous phase was precipitated by addition of an equal volume of isopropanol at room temperature, followed by centrifugation at 15000 rpm at 4°C for 10 min. The DNA pellet was washed with 70% ethanol, air-dried, suspended in 100 μl water, and quantified by a Thermo Fisher Nanodrop instrument.

Plasmid copy number was determined using droplet digital PCR (ddPCR) according to Jahn et al. [24] with minor modifications. Primers and MGB (minor grove binding) probes were designed using Primer Express 3.0 (Thermo Fisher Scientific, www.thermofisher.com). The plasmid-specific Taqman assay was designed to target a β-glucuronidase (GUS) expression cassette included in all plasmids. The native *Agrobacterium* gene *lipA* (GenBank AE007869.2) was used as a chromosomal reference template. The *gusA* and *lipA* probes were labeled with FAM and VIC fluorophores, respectively. The primers 5’ AGCCTTCCACGCCTTTCCT 3’ (forward) and 5’ CCGCTTTTCCCGACGAT 3’ (reverse) were used to amplify a *lipA* short sequence, and the MGB Taqman probe VIC-5’ CCACCTTGCAGATTG 3’-MGB was used for real time *lipA* amplification. The primers 5’ ACCGAATACGGCGTGGATAC 3’ (forward) and 5’ TCCAGCCATGCACACTGATAC 3’ (reverse) were used to amplify a *gusA* short sequence, and the MGB Taqman probe 6FAM-5’ TGTACACCGACATGTGG 3’-MGB was used for real time *gusA* amplification. Duplex PCR reactions were performed following the manufacturer’s recommendations [25]. The 20 μl reaction volume included 0.24 pg/μl total template DNA. The concentrations of each primer and probe were 0.9 μM and 0.25 μM, respectively. Following an initial denaturation step at 95°C, a two-step amplification cycle (94°C and 59°C) was repeated 40 times. Four technical replicates were taken for each DNA sample. Template concentrations were calculated by the Quantasoft 1.4 software (Bio-Rad, www.bio-rad.com) and were given as copy/μl values. Plasmid copy numbers were calculated by normalizing the *gusA* template concentrations to the *lipA* template concentrations.

### Canola transformation

The pMON83937 RepB^Y299H^ mutation plasmid (designated as pMON138207), the original oriRi binary vector pMON83937, and the RK2 oriV binary vector pMON67438 were transferred into *Agrobacterium tumefaciens* ABI (a disarmed nopaline-type strain with the C58 chromosomal background) in a 1 mm gap cuvette by electroporation using a Bio-Rad MicroPulser at 2 kV, and the plasmid-containing bacteria were selected using 50 mg/L spectinomycin and 50 mg/L kanamycin [9]. Canola (*Brassica napus* L, cultivar Ebony) transformation, CP4 transgene copy number determination, and statistical analysis have been described previously [9].

### Statistical analysis

All experiments were carried out under identical conditions and repeated at least three times. Canola explants were randomized before *Agrobacterium* inoculation. Data are presented as averages of the mean of three independent experiments with standard deviation. Significant differences among the results were assessed by SAS with PRO GLIMMIX procedure for plasmid copy number, Analysis of Variance (ANOVA), and/or a Generalized Linear Model for bacterial growth and canola transformation.

## Results and discussion

### pTF::Ri RepB protein sequence has a mutation in a highly conserved residue at the C-terminus

**Fig 1:**
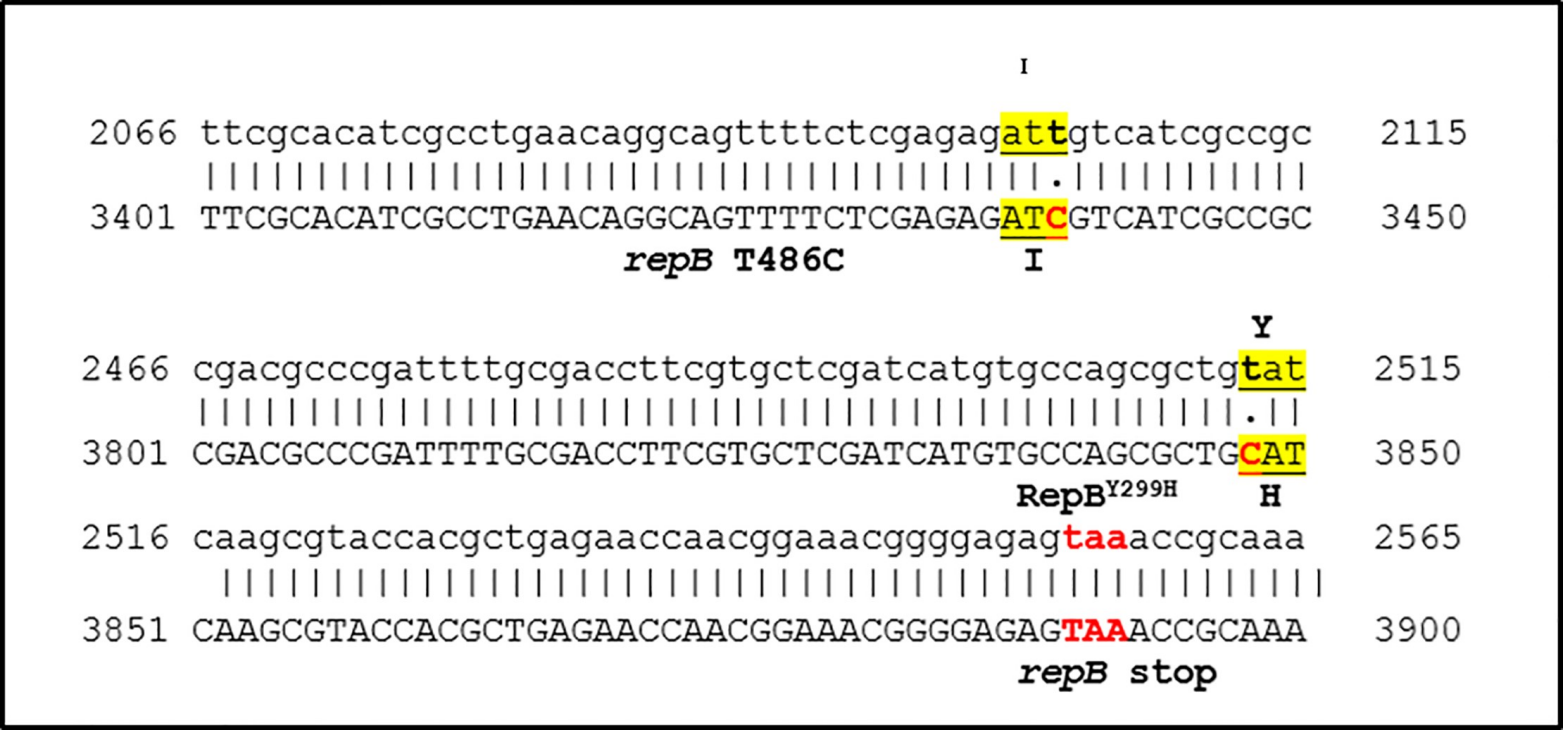
Sequence alignment between part of the genes encoding wild-type *repB* and *repB* from the pTF::Ri binary vector. The T to C changes are highlighted. The *repB* stop codon is marked in red. Upper strand: wild-type *repB* from GenBank X04833; lower strand: pTF::Ri sequence.

Alignment of the pTF::Ri sequence with that of pRi-*repABC* (GenBank # X04833) indicates that there are two T to C point mutations in the *repB* gene of the oriRi: one a T to C silent mutation at 486 bp of the *repB* coding region *(repB* T486C*)*, and the other a T to C point mutation at 895 bp of the *repB* gene leading to a Y299H mutation (RepB^Y299H^; see the *repB* alignment data in S1 Fig). The RepB^Y299H^ mutation results in a change from a hydrophobic to a hydrophilic amino acid in the C-terminal region (Fig 1).

**Fig 2:**
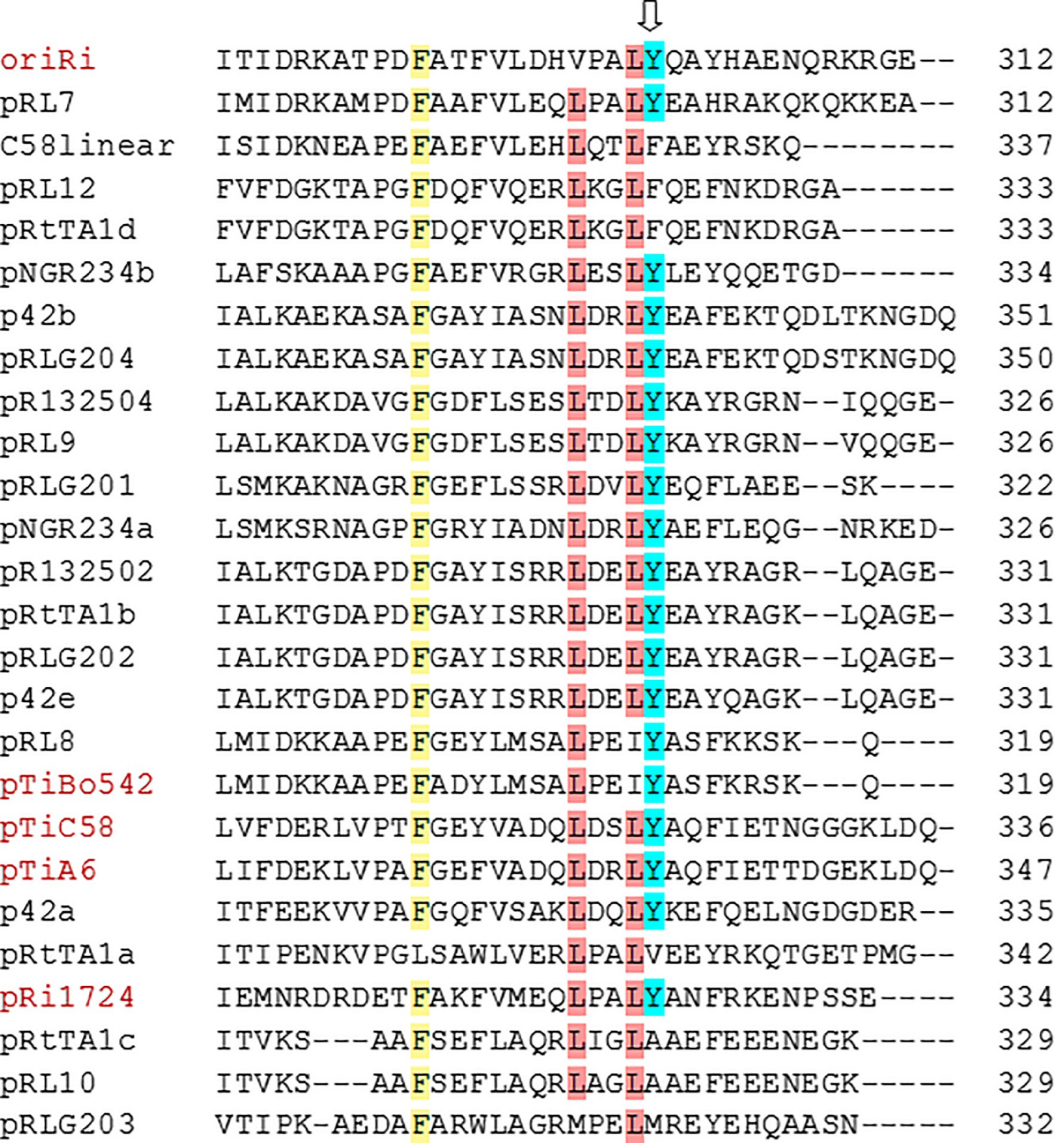
Alignment of the oriRi RepB C-terminus with other RepB C-termini. Arrow indicates the Y299H point mutation. The number at right indicates the last amino acid of each RepB protein. (GenBank accession: oriRi, # X04833; pRL7, *Rhizobium leguminosarum* bv. *viciae* plasmid #AM236081; C58linear, *Agrobacterium tumefaciens* str. C58 linear chromosome # AE007870.2; pRL12, *Rhizobium leguminosarum bv*. *viciae* plasmid pRL12 # AM236086; pRtTA1d, *Rhizobium leguminosarum bv*. *trifolii* strain TA1 plasmid # FJ592234; pNGR234b, *Sinorhizobium fredii* NGR234 plasmid pNGR234b # CP000874; p42b, *Rhizobium etli* CFN 42 plasmid p42b # NC_007763; pRLG204, *Rhizobium leguminosarum bv*. *trifolii* WSM2304 plasmid pRLG204 # NC_011371; pR132504, *Rhizobium leguminosarum bv*. *trifolii* WSM1325 plasmid pR132504; pRL9, *Rhizobium leguminosarum bv*. *viciae* plasmid pRL9 # NC_008379; pRLG201, *Rhizobium leguminosarum bv*. *trifolii* WSM2304 plasmid pRLG201; pNGR234a; *Sinorhizobium fredii* NGR234 plasmid pNGR234a # U00090; pR132502, *Rhizobium leguminosarum bv*. *trifolii* WSM1325 plasmid pR132502; pRtTA1b, *Rhizobium leguminosarum bv*. *trifolii* strain TA1 plasmid pRtTA1b # FJ592235; pRLG202, *Rhizobium leguminosarum bv*. *trifolii* WSM2304 plasmid pRLG202 3 # NC_011366; p42e, *Rhizobium etli* CFN 42 plasmid p42e # NC_007765; pRL8, *Rhizobium leguminosarum bv*. *viciae* plasmid pRL8 # NC_008383; pTiBo542, *Agrobacterium tumefaciens* Ti plasmid pTiBo542 # DQ058764; pTiC58, *Agrobacterium tumefaciens* str. C58 Ti plasmid # NC_003065; pTiA6, *Agrobacterium tumefaciens* octopine-type Ti plasmid # AF242881; p42a, *Rhizobium etli* CFN 42 plasmid p42a # CP000134; pRtTA1a, *Rhizobium leguminosarum bv*. *trifolii* TA1 plasmid pRtTA1a # HM032068; pRi1724, *Agrobacterium rhizogenes* plasmid pRi1724 # NC_002575; pRtTA1c, *Rhizobium leguminosarum bv*. *trifolii* TA1 plasmid pRtTA1c # EU555187; pRL10, *Rhizobium leguminosarum bv*. *viciae* plasmid pRL10 # NC_008381; pRLG203, *Rhizobium leguminosarum bv*. *trifolii* WSM2304 plasmid pRLG203 # NC_011370). Yellow, red, and blue shading indicate generally conserved amino acids.

We aligned 26 RepB C-termini from GenBank (Fig 2). The oriRi RepB Y299 is conserved in the majority of *repABC* replicons, indicating that it may play an important role in RepB function.

### The RepB^Y299H^ mutation increases plasmid copy number in *Agrobacterium* and enhances culture growth

**Fig 3:**
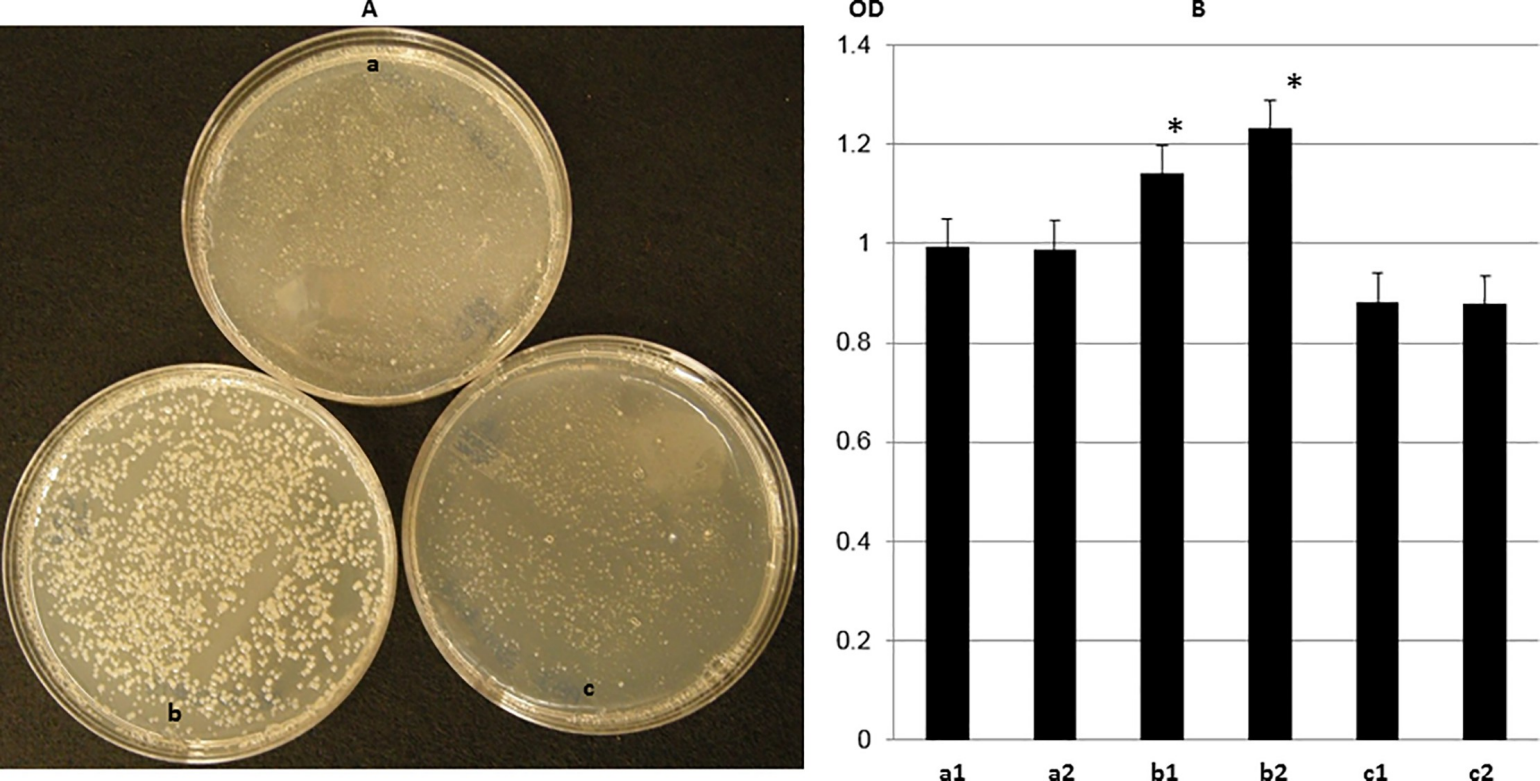
**Growth of *A*. *tumefaciens* with a) pMON83937, WT-RepB; b) pMON83937-RepB**^**Y299H**^**, and c) pMON83937-*repB***^**T486C**^ **silent mutation.** Panel A) Colony size on solidified medium. Panel B) Growth rate in liquid medium (shown as OD at A_600_ on the Y axis): a1, a2; two colonies from pMON83937, b1, b2; two colonies from pMON83937-RepB^Y299H^, c1, c2, two colonies from pMON83937-*repB*^T486C^.

The *repABC* sequence in the oriRi replicon of pMON83937 used in a prior study [9] is identical to that of the oriRi GenBank reference sequence. To address the question of whether the presence of either or both mutations leads to the increase in plasmid copy number of the pTF::Ri plasmid in *Agrobacterium* [21], we carried out site-directed mutagenesis to recapitulate the T to C base changes at 486 bp and 895 bp in pMON83937. These two plasmids were designated pMON83937-*repB*^T486C^ and pMON83937-RepB^Y299H^, respectively. Both plasmids and the control pMON83937 were separately introduced into the nopaline-type strain *Agrobacterium tumefaciens* ABI [9] by electroporation, and the cultures were spread on LB medium containing 50 mg/L spectinomycin and 10 mg/L gentamicin. After 72 hours, colonies harboring pMON83937-RepB^Y299H^ were much larger than those containing the control plasmid or the pMON83937-*repB*^T486C^ silent mutation (Fig 3A). The faster growth could result from a gene dose effect of the elevated plasmid copy number, providing a growth advantage from the multiple copies of the *aadA* gene as increased plasmid copy number [26], which is consistent with our observation that replacing the original *aadA* promoter with a strong prokaryotic promoter can drastically increase the spectinomycin resistance in *Agrobacterium* with the native oriRi binary vector backbone [27]. To investigate this phenomenon further, at least two individual colonies from two sequence confirmed plasmids per mutation were suspended into 5 ml LB medium with the requisite antibiotics at A_600_ = 0.5, an equal volume of culture was inoculated into 50 ml medium, and the ODs recorded after overnight growth (21 hrs). Over three repetitions of this experiment, the cultures with the pMON83937-RepB^Y299H^ mutation consistently showed a 20–25% higher culture density (A_600_) than did the control, whereas the A_600_ of the pMON83937-*repB*^T486C^ cultures showed an approximate 10% growth reduction compared to the control (Fig 3B). Cultures containing the RepB^Y299H^ mutation reached A_600_ = 1.0 about 3 hours earlier than did the control culture.

These observations may indicate an increase in plasmid copy number resulting from the pMON83937-RepB^Y299H^ mutation which confers a growth advantage, and inspired us further to estimate the plasmid copy number against a chromosome reference marker.

**Fig 4:**
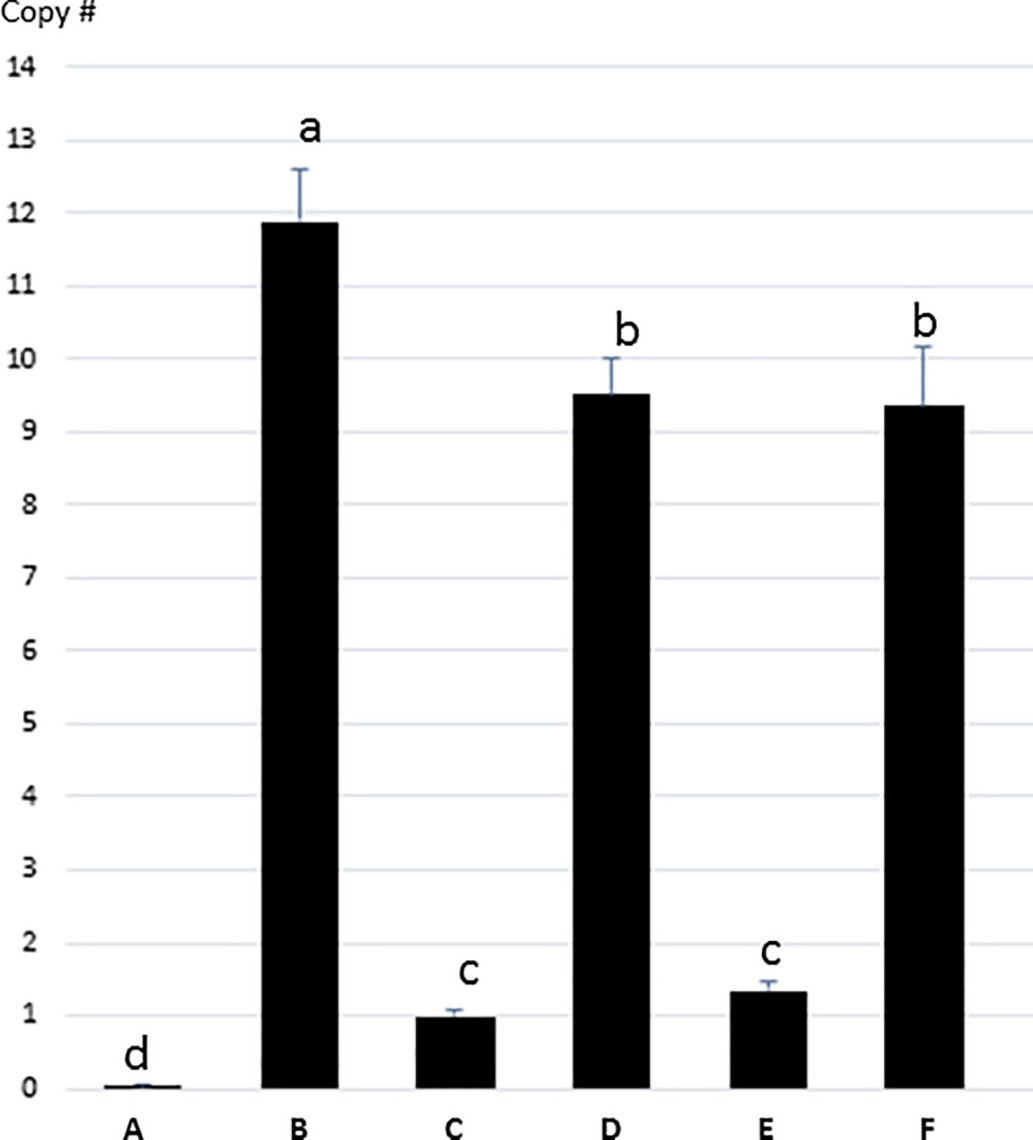
*Agrobacterium* plasmid copy number determination by ddPCR. A) *A*. *tumefaciens* no binary plasmid control; B) pMON67438 oriV replicon; C) pMON83937 oriRi native *repB*; D) pMON83937-RepB^Y299H^; E) pMON83937-*repB*^T486C^; F) pMON83937-RepB^Y299H^ + *repB*^T486C^ double mutations. Error bars are shown in the diagram. * B, D and F are significant higher copies than C (native oriRi) and E (T486C silent mutation) at P = 0.05. B (RK2 oriV) is also significantly higher than D and F (the oriRi mutants). Small letters a, b, c and d above the bars indicate statistically significantly different groups (data and statistical analysis in S1 Table).

To determine plasmid copy number in *Agrobacterium*, a DNA-based Taqman assay was employed using the single copy *Agrobacterium* chromosomal gene *lipA* as an internal copy number control. Atu0972 (*lipA*) was chosen as the internal control for comparison to the *gusA* sequence in the binary vectors because *lipA* copy number does not vary upon acetosyringone treatment [28]. A third *repB* double mutation plasmid, pMON83937 RepB^Y299H^ + *repB*^T486C^, was produced for copy number determination. As shown in Fig 4, the plasmid copy numbers varied between 1 (pMON83937, native oriRi) and 12 (pMON67438, multi-copy oriV binary vector) per cell. The oriRi RepB^Y299H^ mutant and the RepB^Y299H^ and *repB*^T486C^ double mutants were estimated to contain 9–10 copies/cell (Fig 4D and 4F), whereas the oriRi *repB*^T486C^ silent mutation alone showed approximately 1 copy/cell (Fig 4E).

The *trans*-acting RepA and RepB proteins of the *repABC* plasmid resemble ParA and ParB partitioning proteins. The RepB proteins bind the cis-acting centromere-like *parS*, and all three elements are essential for *repABC* plasmid partitioning [18, 19, 20, 29]. In the ParAB system, ParB proteins bind *parS* to form a partition complex which recruits the ParA ATPase to form a segrosome and activate the ATPase for DNA segregation [30]. RepB proteins can form dimers/multimers, and the dimerization domain is located at the C-terminus of RepB from *Rhizobium leguminosarum* [18], which is conserved among the *repABC* family (Fig 2). The RepB with a C-terminus deletion was not able to bind DNA in a gel shift assay, suggesting that lack of dimerization prevents DNA binding of RepB [18]. The increased copy number of the oriRi with the RepB^Y299H^ mutation in *Agrobacterium* may result from decreased RepB^Y299H^ binding affinity to the the *parS* site, which prevents forming a partition complex and may lead to the appearance of plasmid multimers. It is believed that plasmid multimers may be formed during low copy plasmid replication, requiring action of a multimer resolution system to convert them into monomers through site-specific recombination [14]. Transcription of *repABC* genes is also regulated by the RepA autorepressor, and RepB enhances RepA binding to the P4 promoter motif [31]. It is also possible that the RepB^Y299H^ mutation in the oriRi plasmid reduces RepA-RepB cooperative binding to the *repABC* operon promoter due to diminished RepB^Y299H^ dimerization, leading to disturbances in transcriptional autorepression exerted by RepAB. The plasmid copy number increase by the inducer is not fully understood yet [31].

The stability of the *repABC* replicon with the RepB^Y299H^ mutation was not determined because our plant transformation experiments were carried out with antibiotic selection, as was the case in previous experiments by Oltmanns et al. [21]. It is possible that the mutation affects dimerization/oligomerization, which partially blocks plasmid partitioning and leads to the accumulation of higher plasmid copy numbers in cells. Plasmid-free *Agrobacterium* cells may be avoided or minimized because the *Agrobacterium* used for plant transformation was grown using antibiotic selection.

The difference in plasmid copy number between Oltmanns et al. [21] and this study may result from the assay method, *Agrobacterium* strain, or growth time difference. Plasmid copy numbers are determined primarily by their replication origins, which show significant cell-to-cell variation within every population [32]. Oltmanns et al. [21] reported 15–20 copies/cell with pTF::Ri carrying RepB^Y299H^ and *repB*^T486C^ mutations in *A*. *tumefaciens* EHA105 using a DNA blot assay, whereas in this study we estimated that both the single and the double mutations in a nopaline-type ABI strain have 9–10 copies/cell, using a ddPCR assay. The DNA blot method likely has less resolution. The control vector RK2 oriV vector was estimated to be 7–10 copies/cell by DNA blot [21] and 12 copy/cell by ddPCR in this study. In previous work we estimated that the copy number of the control RK2 oriV vector in ABI strain was 4–8 times higher than that of the native oriRi based vector using DNA blot analysis [9]. The RK2 oriV copy number determined by ddPCR is close to the RK2 oriV-containing plasmid pSoup which was determined by a real time PCR method [10].

### The wild-type oriRi outperforms the RepB^Y299H^ mutated oriRi for generating single copy, backbone-free transgenic events in canola transformation

**Fig 5:**
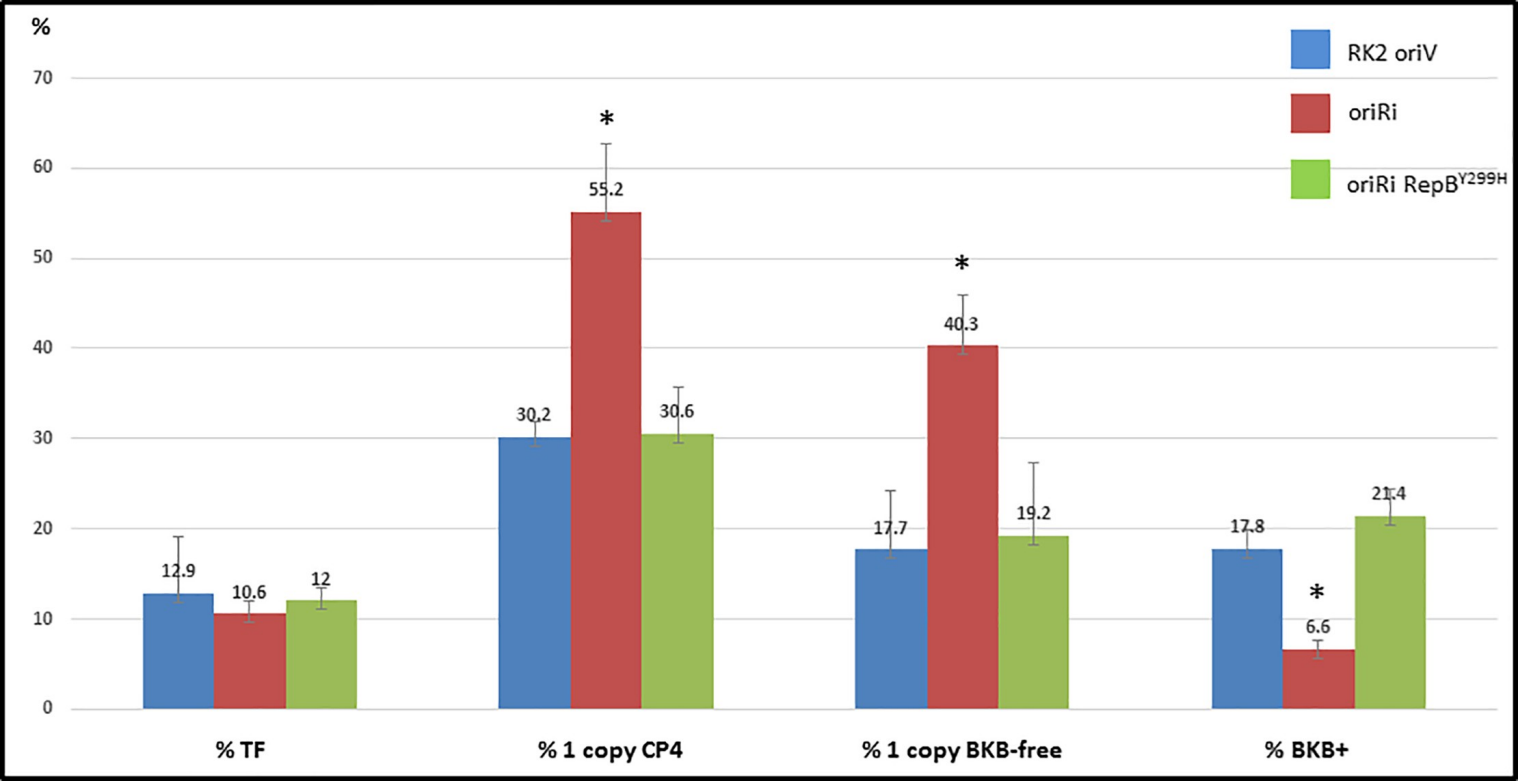
Effect of the oriRi with RepB^Y299H^ mutation on quality transgenic canola plant production. Total oriV (pMON67438) events, n = 421; total oriRi (pMON83937) events, n = 379; total oriRi RepB^Y299H^ (pMON138207) events n = 462. Error bars are shown in the diagram. *significant difference from the RK2 oriV and the oriRi RepB^Y299H^ at P = 0.01. TF: transformation frequency (transgenic events/explant number); 1 copy CP4: single copy CP4 positive frequency; 1 copy CP4 BKB-free: single copy CP4 positive, backbone-free transgenic plant frequency; BKB+: backbone positive transgenic plant frequency. Raw data and statistical analyses in S2 Table for the canola transformation.

Due to the contradictory observations in single copy, backbone-free plant production between the two versions of oriRi binary vectors [9,21], we speculated that the higher copy number of pTF::Ri [21] with the RepB^Y299H^ mutation abolished the advantage of the native oriRi binary vector for production of single copy transgenic plants. To test this hypothesis, we compared the wild-type oriRi binary vector (pMON83937) and the RepB^Y299H^ mutant (pMON138207), as well as the multi-copy RK2 oriV based vector (pMON67438), in canola transformation. In total, 1262 transgenic canola events were generated for all three constructs, and all three constructs showed statistically equivalent transformation frequencies, which is defined as the number of independent transformed plants produced per number of explants used (Fig 5). Both the single copy and the single copy, backbone-free transgenic plant frequencies were equivalent between the multi-copy RK2 oriV based vector and the oriRi vector with RepB^Y299H^ high copy mutant, whereas the native single copy oriRi vector showed a significant improvement in the frequency of plants with a single copy of the transgene and absence of vector backbone sequences. The frequency of plants containing vector backbone sequences was decreased three-fold using the native oriRi vector compared to the RK2 oriV and the high copy oriRi mutant vectors.

This canola transformation result is consistent with the earlier observation that the high copy pTF::Ri resulted in a similar frequency in single copy, backbone-free plants to the multi-copy RK2 oriV binary vectors [21]. Our results support the hypothesis that T-DNA border processing involves a stoichiometric reaction between the endonuclease VirD2 and the border elements, and an excess of borders may titrate-out VirD2, resulting in incomplete processing [9, 33]. This hypothesis is also supported by experiments in which a T-DNA was integrated as a single copy into an *Agrobacterium* chromosome, which drastically decreased *Agrobacterium* chromosome integration frequency in transgenic plants compared to other multi-copy binary vectors [21].

## Conclusion

Our data revealed that the RepB C-terminus is critical for copy number control in a *repABC* replicon, which may be related to plasmid partitioning [18]. A single nucleotide mutation at RepB^Y299H^ in the pRi-*repABC* derived plant transformation binary vectors is responsible for an increase in plasmid copy number in *Agrobacterium*, and a concomitant decrease in relative frequency of single copy, backbone-free transgenic plants produced. This result also cautions biotechnological researchers that sequence confirmation is required for critical experiments and applications, because a single nucleotide mutation could lead to a very different conclusion.

## Supporting information

S1 Fig RepB nucleic acid, protein sequences and mutations. (DOCX)

S1 Table Plasmid copy number determination by ddPCR and statistical analysis. (XLSX)

S2 Table OriRi mutant canola transformation raw data and statistical analysis. (XLSX)
