# Supplementary material for: RepB C-terminus mutation of a pRi-repABC binary vector affects plasmid copy number in Agrobacterium and transgene copy number in plants
Source: PLoS One. 2018 Nov 9;13(11):e0200972. doi: 10.1371/journal.pone.0200972 (PMC6226153; doi:10.1371/journal.pone.0200972)
Supplement: S1 Fig — (DOCX) [file pone.0200972.s001.docx]

**S1 Fig: RepB nucleic acid, protein sequences and mutation positions**

atgccgctcctcggcgtaacaaggaaggagcgcgatccggcaacgaagctcacagcgaac

M P L L G V T R K E R D P A T K L T A N

attggtaacgcactgcgagagcaaaacgatcgtcttagccgtgccgaagagatcgagcgg

I G N A L R E Q N D R L S R A E E I E R

cgtctcgctgaaggtcaggcagtgatagagttggatgcctcgtcaatagaaccgtctttc

R L A E G Q A V I E L D A S S I E P S F

gtgcaggatcgtatgcgaggggacattgacgggctccttacttcgatccgggaacaagga

V Q D R M R G D I D G L L T S I R E Q G

cagcaagtcccaatccttgtgcgaccgcatccgagccagccgggccgatatcaggttgcc

Q Q V P I L V R P H P S Q P G R Y Q V A

ttcggccaccgccggctacgcgccgtttcagaactcggacttccggtcagagcggtcgtt

F G H R R L R A V S E L G L P V R A V V

cgcgaactgacggacgagcaagtggtcgtagcacagggtcaggaaaacaatgagcgcgaa

R E L T D E Q V V V A Q G Q E N N E R E

gatcttaccttcatcgaaaaggcgcgcttcgcacatcgcctgaacaggcagttttctcga

D L T F I E K A R F A H R L N R Q F S R

gagattgtcatcgccgcgatgtcgatcgacaagagcaatttgtccaagatgcttctgctc

E I V I A A M S I D K S N L S K M L L L

gtcgacgccctcccctctgaactgaccgatgctattggtgccgctcctggtgttggacgg

V D A L P S E L T D A I G A A P G V G R

ccgagttggcaacaacttgccgagctgattgagaaagtttcttcaccggccgacgtggct

P S W Q Q L A E L I E K V S S P A D V A

aaatatgctatgtcggaggaagttcaagcgctgccatcggcagaacgattcaaggcggtg

K Y A M S E E V Q A L P S A E R F K A V

atcgctagtctgaagcccagtcgggttgcgcgtggacttcccgaggtcatggccacccca

I A S L K P S R V A R G L P E V M A T P

gacggcaccagaattgcacaggtgacgcagagcaaggccaaactggaaatcacgattgac

D G T R I A Q V T Q S K A K L E I T I D

aggaaggcgacgcccgattttgcgaccttcgtgctcgatcatgtgccagcgctgtatcaa

R K A T P D F A T F V L D H V P A L Y Q

gcgtaccacgctgagaaccaacggaaacggggagagtaa

A Y H A E N Q R K R G E -
